# Cellular and humoral immune responses against the *Plasmodium vivax* MSP-1_19_ malaria vaccine candidate in individuals living in a Brazilian endemic area in northeastern Amazon

**DOI:** 10.1186/1475-2875-13-S1-P32

**Published:** 2014-09-22

**Authors:** Evelyn Kety Pratt Riccio, Paulo Renato Rivas Totino, Irene Silva Soares, Maurício Martins Rodrigues, Cláudio Tadeu Daniel Ribeiro, Maria de Fátima Ferreira da Cruz

**Affiliations:** 1 Fundação Oswaldo Cruz, Rio de Janeiro, RJ, Brazil; 2 Universidade do Estado de São Paulo, São Paulo/SP, Brazil

## Background

*Plasmodium vivax* Merozoite Surface Protein-1 (MSP-1) is an antigen considered to be one of the leading malaria vaccine candidates. PvMSP-1 is immunogenic and evidences suggest that it is a target for protective immunity against asexual blood stages of malaria parasites. This study aims to evaluate the acquired cellular and antibody immune responses against PvMSP-1 in individuals exposed to malaria infections in a Brazilian malaria endemic area.

## Methods

The study was carried out in Paragominas, Pará State, in the Brazilian Amazon. Blood samples were collected from 35 individuals with uncomplicated malaria. Peripheral blood mononuclear cells were isolated and the cellular proliferation and activation was analyzed in presence of 19 kDa fragment of MSP-1 (PvMSP-1_19_) and *P. falciparum* PSS1 crude antigen. Antibodies IgE, IgM, IgG and IgG subclass and the levels of TNF-α, IFN-γ and IL-10 were measured by enzyme-linked immunosorbent assay (ELISA).

## Results

The prevalence of activated CD4^+^was greater than CD8^+^ T cells, in both ex-vivo and in 96 h culture in presence of PvMSP-1_19_ and PSS1 antigen. A low proliferative response against PvMSP-1_19_ and PSS1 crude antigen after 96 h culture was observed. High plasmatic levels of IFN-g and IL-10 as well as lower TNF levels were also detected in malaria patients. However, in the 96 h supernatant culture, the dynamics of cytokine responses differed from those depicted on plasma assays; in presence of PvMSP-1_19_ stimulus, higher levels of TNF were noted in supernatant 96 h culture of malaria patients’ cells while low levels of IFN-g and IL-10 were verified. High frequency of malaria patients presenting antibodies against PvMSP-1_19_ was evidenced, regardless of class or IgG subclass. PvMSP-1_19_-induced antibodies were predominantly on non-cytophilic subclasses.

## Conclusions

PvMSP-1_19_ was able to induce a high cellular activation, leading to production of TNF and emphasizes the high immunogenicity of PvMSP-1_19_ in naturally exposed individuals and therefore its potential as a malaria vaccine candidate.

